# Auf dem Weg zur „Einheit“: Józef Chałasiński und die Suche nach einer „erlaubten“ Genealogie der Soziologie im Nachkriegspolen (1945–1951)

**DOI:** 10.1007/s00048-020-00267-3

**Published:** 2020-08-04

**Authors:** Aleksei Lokhmatov

**Affiliations:** EUmanities Fellow, Philosophische Fakultät, Universität zu Köln, Aachener Str. 217, 50931 Köln, Deutschland

**Keywords:** Geschichte der Sozialwissenschaften, Polnische Soziologie, Stalinisierung, Józef Chałasiński, Erster Kongress der polnischen Wissenschaft, Lemberg-Warschau-Schule, History of social sciences, Polish sociology, Stalinisation, Józef Chałasiński, First Congress of Polish Science, Lvov-Warsaw School

## Abstract

Dieser Aufsatz beschäftigt sich mit den öffentlichen Debatten über die Genealogie der polnischen Sozialwissenschaften nach dem Zweiten Weltkrieg. Es wird gezeigt, wie sich in der Periode zwischen dem Ende des Zweiten Weltkrieges (1945) und der Stalinisierung der polnischen Wissenschaft im Rahmen des „Ersten Kongresses der polnischen Wissenschaft“ (1951) die Grenzen des Erlaubten in öffentlichen Diskussionen über die wissenschaftliche Identität der Soziologie verschoben haben. Der Artikel, der vor allem auf der Analyse der öffentlichen Haltung des Organisators der soziologischen Gruppe Józef Chałasiński basiert ist, beschreibt die Entwicklungsetappen des öffentlichen Diskurses über die akademische Tradition der Sozialwissenschaften, die den zunehmenden Einfluss der politischen Agenda auf die wissenschaftliche Praxis widerspiegeln. Obwohl unmittelbar nach dem Krieg die Kontinuität mit der Zwischenkriegstradition und der „limitierte Charakter“ des marxistischen Ansatzes betont wurden, hat die beginnende Stalinisierung des öffentlichen Raums 1948 dazu geführt, dass die politische Rolle der Soziologie, die zusammen mit dem Marxismus dem progressiven Lager angehörte, zum zentralen Punkt ihrer eigenen Genealogie wurde. Die Schließung der soziologischen Abteilungen und Zeitschriften zwischen 1950 und 1951 markierten dabei die neue Etappe im Rahmen der Vorbereitungssitzungen zum ersten wissenschaftlichen Kongress. Eine der Hauptaufgaben für die Sozialwissenschaften im Rahmen dieses Kongresses bestand darin, gemeinsam mit den Philosophen eine progressive wissenschaftliche Tradition Polens zu konstruieren. Der Kongress wurde für die polnischen Wissenschaftler zur Aufgabe mit bekanntem Ergebnis und mehreren obligatorischen Elementen wie einer „fortschrittlichen nationalen Tradition“, dem „Marxismus-Leninismus“ und einem „Sechsjahresplan“ zu verfassen. Trotz des starken Drucks von Seiten der Politik konnten die Teilnehmer der Diskussionen keinen Kompromiss im Hinblick auf die progressive Tradition der polnischen Sozialwissenschaften finden und so die notwendige „Einheit“ erreichen.

## Abstract (en)

This article deals with the public debates on the genealogy of Polish social sciences after the Second World War. The author shows how the changes in political conditions in the period between the end of the war (1945) and the ‘Stalinisation’ of Polish science at the First Congress of Polish Science (1951) influenced the ‘limits of the permissible’ in public discussions about the scientific identity of sociology. The article describes the stages in the development of public discourse on the genealogy of the social sciences and thematises the increasing influence of the political agenda on the public practices of academics. When, immediately after the war, continuity with inter-war tradition, and the limited character of the Marxist approach, were particularly emphasised in the public discourse on the genealogy of social sciences, the beginning of the ‘Stalinisation’ of public space in 1948 forced the scholars to put emphasis on the political role of sociology, which, according to them, belonged to the ‘progressive camp’ together with Marxism. The closing of the sociological departments, and a journal, between 1950 and 1951 started a new stage of the debates within the preparatory meetings for the scientific congress. At this congress, the task of the social scientists was to construct, together with philosophers, a ‘progressive scientific tradition’ in Poland. For the Polish social scientists, the congress was a task to write a ‘mathematical equation’ with a known result and several mandatory elements such as ‘the progressive national tradition,’ ‘Marxism-Leninism,’ and ‘six-year plan.’ However, even under strong political pressure, the participants in the discussions did not come to a compromise concerning the progressive tradition of the Polish social sciences, and did not achieve the necessary ‘unity.’

Eine Historisierung der Wissenschaften ist kaum denkbar, ohne die jeweiligen sozialen und politischen Bedingungen des Erkenntnisgewinns sowie die Kommunikation der betreffenden Wissenschaftler zu berücksichtigen. Die Tatsache, dass diese einführende Bemerkung recht trivial erscheint, zeigt, wie stark die Entwicklung der Wissenschaftsgeschichte in den letzten Jahrzehnten die aktuellen Paradigmen der Geschichtsschreibung beeinflusst hat. Die Idee, dass die Wissenschaftler selbst wissenschaftliche Tatsachen konstruieren und kollektiv Regime der Relativität für diese Tatsachen schaffen (Fleck 1935), hat später dazu geführt, dass die historische Entwicklung der Wissenschaften als Abfolge wechselnder Paradigmen vorgestellt wurde (Kuhn 1962). Parallel dazu hat die Wissenssoziologie eine gesellschaftlich orientierte Perspektive auf Wissenschaft entwickelt (Mannheim 1931), die die Wissenschaftsgeschichte sehr stark mit gesellschaftlichen Prozessen verbunden hat. Aus dieser soziologischen Perspektive wurde Wissenschaftsgeschichte häufig als eine Geschichte der wissenschaftlichen Institutionen dargestellt (Ringer 1969), eine Sichtweise, die bis heute eine wichtige Rolle in der wissenschaftshistorischen Praxis innehat (Fleck et al. 2019). Zugleich ist die *historical epistemology *zum Forschungsprogramm geworden, das einen theoretischen Rahmen für Wissensgeschichte schuf, in dem Wissenschaft in einem breiteren ideen- und sozialgeschichtlichen Kontext betrachtet wird (Daston 1994).

Mit diesen wenigen Worten soll die wechselseitige Beziehung zwischen sozialen Prozessen und der Entwicklung der Wissenschaft angerissen werden, um den Blickwinkel sodann auf jene Fälle zu richten, in denen das politische Feld die Autonomie der Wissenschaft negierte. Im 20. Jahrhundert schränkten unterschiedliche politische Regime die „Freiheit der Forschung“ ein. Die Beziehungen zwischen politischer Agenda und wissenschaftlicher Praxis sowie die Rolle von Wissenschaftlern in der Formation nationaler, geografischer und politischer Realität haben zunehmend das Interesse von Fachhistoriker*innen geweckt und sind in den letzten Jahren zu einem zentralen Thema der Historiographie geworden.^1^ Die Sowjetunion wurde dabei oft als Beispiel staatlicher Regulierung von Forschungsprogrammen aller wissenschaftlichen Disziplinen angeführt, die nach den ideologischen Richtlinien des Stalinismus zur Entwicklung der Volkswirtschaft beitragen sollten. Die Geistes- und Sozialwissenschaften wurden im Zuge repressiver ideologischer Maßnahmen der 1930er Jahre offiziell auf den „einzig richtigen Ansatz des Marxismus-Leninismus“ reduziert. Im Vergleich mit den revolutionären wissenschaftlichen Projekten der 1920er Jahre traten zunehmend nationalistische Züge hervor. Alle Teilrepubliken der Sowjetunion sollten ihre nationalen wissenschaftlichen Traditionen als die Errungenschaften der „besten Söhne und Töchter ihrer Völker“ vorstellen. Die Schaffung fortschrittlicher nationaler Traditionen war, unter anderem, ein wichtiges Instrument zur Vereinigung wissenschaftlicher Ansichten und zur Ausarbeitung „legitimer“ Vorbilder für die wissenschaftliche Arbeit (Krementsov 1997; Pollock 2006).

Der Zweite Weltkrieg und die nach ihm entstandene neue Weltordnung haben politische Bedingungen geschaffen, die begünstigten, dass das stalinistische Modell der Wissenschaft in der Einflusssphäre der Sowjetunion verbreitet werden konnte (David-Fox & Peteri 2000). Polen gehörte nach dem Krieg zu jenen Ländern, die im sowjetischen Einflussbereich lagen, ihre formale staatliche Unabhängigkeit allerdings nicht verloren hatten (Kersten 1989). Da Stalin und die Sowjetregierung den Widerstand der polnischen Bevölkerung vermeiden wollten, wurde mit der Schaffung polnischer staatlicher Institutionen am Ende des Krieges ein Sonderweg für das Land vorbereitet: Eine „sanfte Revolution“ sollte auf nicht repressive Weise wissenschaftliche Reformen umsetzen, was im neuen polnischen Staat in den ersten Nachkriegsjahren eine gewisse Autonomie der wissenschaftlichen Debatten zur Folge hatte. Im wissenschaftlichen Feld nutzten zahlreiche Gruppierungen die unpräzise formulierte staatliche Ideologie aus, gründeten Zeitschriften und versuchten, verschiedene wissenschaftliche Traditionen der Vorkriegszeit wieder aufzunehmen. (Stobiecki 1993: 35–85; Krasucki 2009: 111–119; Babiracki 2015: 52–96).^2^

Der beginnende Kalte Krieg führte in Polen dann aber zu einer rapiden „Stalinisierung“ des öffentlichen Raums und damit auch der wissenschaftlichen Debatten. Auf politischer Ebene wurde die Gründung des kommunistischen Informationsbüros (Kominform) 1947 zum Zeichen dafür, dass die Sowjetunion keine „Sonderwege“ mehr unter den sozialistischen Ländern tolerieren würde. Auf der ersten Konferenz des Kominform, die im polnischen Städtchen Szklarska Poręba stattfand, sagte der Hauptideologe der sowjetischen Regierung Andrei Schdanow, dass „die intentionale (*narotschitoe*) Betonung der Unabhängigkeit von Moskau in die Hände der Feinde spiele“, und es notwendig sei, die Einheit zwischen den kommunistischen Parteien zu demonstrieren (Adibekov 1998: 300). Ab 1948 wurde in Polen der Prozess der „Vereinheitlichung“ des öffentlichen Raums begonnen, der den wissenschaftlichen Alltag stark beeinflusste (Connelly 2000; Górny 2013).

Der erste Kongress der polnischen Wissenschaft (Juli 1951) sowie die Gründung der Polnischen Akademie der Wissenschaften (Oktober 1951) waren zwei wichtige Schritte auf dem Weg der Angleichung des akademischen Systems Polens an das stalinistische Wissenschaftsmodell. Es war Chopins „heroische“ Polonaise As-Dur op. 53, die ausgewählt wurde, um den Fernsehfilm über die Eröffnung des „Ersten Kongresses der polnischen Wissenschaft“ im Sommer 1951 zu begleiten. Das Warschauer Polytechnikum (der Sitz des Kongresses) wurde von polnischen Filmemachern als Zentrum der Aufklärung und des Fortschritts im wiederauferstandenen polnischen Staat dargestellt (Wytwórnia Filmów Dok 1951). Die Gesichter von Nikolaus Kopernikus und Maria Skłodowska-Curie schmückten die Briefmarken des Jahres und sollten auf feierliche Weise die „wissenschaftlichen Errungenschaften der polnischen Nation“ verkörpern sowie die „glorreiche nationale Tradition Polens“ ehren. Der polnische Premierminister Józef Cyrankiewicz begrüßte das akademische Publikum auf dem Kongress mit folgenden Worten: „Die wundervollen Traditionen ihrer verehrten Vorgänger fortsetzend, der Nation und Polen dienend, um dadurch dem Fortschritt und der Menschheit [zu dienen], soll die polnische Wissenschaft zum Schöpfer der Größe des polnischen Volkes“ werden.^4^ Auf diese Weise wurde die Proklamierung der angewandten Wissenschaft, die das Leitmotiv des Kongresses geworden war, nicht als Bruch mit der Vergangenheit, sondern als Fortsetzung der fortschrittlichen polnischen intellektuellen Tradition dargestellt.

Dieser Wissenschaftskongress hatte zum Ziel, den „einheitlichen Willen“ der polnischen Wissenschaftler zu demonstrieren und ihre wissenschaftliche Arbeit ausschließlich auf den fortschrittlichen Traditionen der polnischen Nation zu gründen. Dennoch erwiesen sich die Versuche, diese „fortschrittliche“ Tradition in den polnischen Sozial- und Geisteswissenschaften zu etablieren, als äußerst problematisch. Die bislang wenig ausgeprägte marxistische Tradition, die Kritik der sowjetisch-marxistischen Philosophen an der Lemberg-Warschau-Schule sowie die in der Sowjetunion als „bürgerliche Wissenschaft“ verbotene Soziologie waren für die Sprecher der „Sektion für Sozial- und Geisteswissenschaften“ des Kongresses ein Problem. Vor dem Hintergrund allgemeiner Einigkeit, die sich in den gedruckten Kongressberichten vermittelt, ist auffällig, dass der offizielle Bericht der Sektion für Geisteswissenschaften des Kongresses den Marxismus-Leninismus zwar lobt, aber keinerlei Einwilligung bezüglich methodischer sowie theoretischer Ansätze in diesen Forschungsfeldern signalisiert.^5^

Diese deutlichen Widersprüche zwischen der polnischen wissenschaftlichen Tradition und der von Seiten der Sowjetunion erwarteten methodischen Einheit schufen einen spezifischen Kontext für die Stalinisierung in Polen. Es liegen inzwischen diverse Studien vor, die veranschaulichen, wie heterogen und gelegentlich auch problematisch dieser Prozess in verschiedenen sozialistischen Staaten verlief (Jessen 1999; Connelly 2000; Górny 2013; Péteri 2019). Im Falle Polens kann nachvollzogen werden, wie wahrnehmbar sich die Bedingungen der wissenschaftlichen sowie öffentlichen Debatten gleich nach dem Krieg und nach der Stalinisierung voneinander unterschieden (Bąbiak 2014: 5–68). Die Stalinisierung der polnischen Wissenschaft folgte dabei aber keinem klaren Plan und war in gewissem Maße ein Versuch des polnischen Regierungsapparates zu erraten, was jeweils vonseiten Moskaus erwartet wurde. Archivmaterialien zu öffentlichen Diskussionen unter Wissenschaftler*innen, Publikationen in Periodika bzw. Protokolle der Vorbereitungstreffen vor dem ersten Wissenschaftskongress 1951 sind wertvolle Quellen zur Untersuchung des Einflusses der rapiden politischen Umwälzungen auf die wissenschaftliche Praxis im Polen der Nachkriegszeit.

Die Verschiebungen der „Grenzen des Erlaubten“ im Nachkriegspolen waren bislang vor allem Gegenstand der historischen Forschung, in erster Linie im Rahmen der Geschichte der Geschichtsschreibung (Stobiecki 1993; Rutkowski 2007; Górny 2013). Innerhalb der Fachgeschichte der Soziologie (Markiewicz 1964: 272–275; Bielecka-Prus 2011: 735–765; Sułek 2020: 314–317), für die dieser Zeitraum eher als Phase wahrgenommen wurde, in der es zu überleben galt (Bucholc 2016: 15–28), spielt diese Periode bislang keine große Rolle, weil sie kaum zur Entwicklung der soziologischen Theorie in Polen beigetragen hat. Das Ziel des vorliegenden Beitrages ist es deshalb, den oft isoliert auf ein bestimmtes Fach bezogenen Ansatz der Geschichtsschreibung in den Geistes- und Sozialwissenschaften, um eine gesellschaftliche und politische Perspektive zu ergänzen. Deswegen steht im Zentrum dieses Aufsatzes die Frage, wie die polnischen Soziologen die historische Identität ihrer Disziplin auf die sich stets verändernde politische Realität anzupassen versuchten. Die Veränderungen in der Repräsentation ihrer wissenschaftlichen Tradition stellte unter anderem einen Versuch dar, durch die Interpretationen der Rolle und der Werke der polnischen Soziologien einen Raum für Soziologie in der akademischen Landschaft Polens unter den immer neuen politischen Bedingungen zu schaffen.

Eine zentrale Figur in den öffentlichen Debatten über die Genealogie der Soziologie war Józef Chałsiński, der spätere Vorsitzende der geisteswissenschaftlichen Sektion des ersten Wissenschaftskongresses. Zwischen 1949 und 1952 war er Rektor der Universität Łódź und ordentliches Mitglied der neugegründeten polnischen Akademie der Wissenschaften (1951). Darüber hinaus war Chałasiński der Hauptorganisator der soziologischen Gruppe in Polen nach dem Zweiten Weltkrieg und vertrat Soziologie als Disziplin auf wissenschaftspolitischer Ebene. Die Veränderungen in seiner öffentlichen Haltung und die damit einhergehenden Reaktionen seiner Opponenten tragen also zentral zum Verständnis der öffentlichen Debatten vor dem Hintergrund politischer Veränderungen bei, weshalb die Person Chałasińskis zum Angelpunkt der folgenden Ausführungen werden soll.

## Kriegsende: Wiederaufbau der Soziologie und Konstruktion ihrer Genealogie

Es war Józef Chałasiński, der die Wiederbelebung der wichtigsten soziologischen Zeitschrift der Zwischenkriegszeit, *Soziologische Rundschau* (*Przegląd socjologiczny*), initiiert hatte. Die Fortsetzung der Zeitschrift, die 1930 von Chałasińskis Lehrer Florian Znaniecki (1882–1958) gegründet worden war, sollte die Kontinuität der polnischen Sozialwissenschaftler mit den vorhergehenden Jahrzehnten demonstrieren. Der erste Artikel der neuen Ausgabe „Wiederaufnahme der soziologischen Zeitschrift“ berichtete über die Leistungen der Zeitschrift, bevor sie ihre Tätigkeit wegen des Krieges hatte einstellen müssen. Die kurze Beschreibung der Verluste der polnischen Soziologie während des Krieges sollte zugleich die Notwendigkeit verdeutlichen, nicht nur das methodologische, sondern auch das ideologische Erbe der großen Persönlichkeiten der polnischen Soziologie unter den neuen politischen Bedingungen zu berücksichtigen (Chałasiński 1946a: 1–3).

Einer der einflussreichsten polnischen Soziologen der Zwischenkriegszeit, der Marxist Ludwik Krzywicki (1859–1941), wurde zur Hauptfigur der von Chałasiński neu konstruierten Genealogie der polnischen Sozialwissenschaften.^6^ Chałasiński legte einen Schwerpunkt auf die sozialen Aktivitäten Krzywickis und dessen Essaysammlung *Sic itur ad virtutem*, die er als ein „Evangelium des polnischen Handelns“ betrachtete und die seiner Meinung nach zu einem „Aufruf an die junge Generation“ werden müsse (Chałasiński 1946a: 2). Die Erforschung der Ideen spielte eine besondere Rolle in dem von Krzywicki entwickelten Konzept des „historischen Prozesses“. Diesem Ansatz folgend führte der historische Materialismus in seiner Überzeugung, dass Ideen mit der Entwicklung von Produktivkräften und der Bildung neuer Klassen verbunden waren, zu dem Befund, dass „das Bewusstsein [der Klassen] zu einem Faktor von größter Bedeutung“ geworden sei. Auf diese Weise bildeten für Krzywicki „Gewohnheiten und Vorurteile, Grundsätze und Überzeugungen, Gefühle und Temperament“ sowie „politische und rechtliche Institutionen, moralische und ästhetische Ansichten“ „eine ganzheitliche Kategorie, den historischen Hintergrund (*podłoże historyczne*)“, der in besonderer Weise zu untersuchen sei.

Dennoch zitierte Chałasiński ausgiebig aus Krzywickis Schriften, die beweisen sollten, dass sich der marxistische Denker der unterschiedlich verlaufenden historischen Prozesse in verschiedenen Ländern bewusst sei. Chałasiński würdigte das Neue an Krzywickis „psychologischer und historischer“ Herangehensweise und betonte insbesondere die Tatsache, dass dieser marxistische Denker die „Grenzen der Anwendbarkeit (*granice stosowalności*)“ des historischen Materialismus erkannt habe. Darüber hinaus bediente sich Chałasiński der politischen und akademischen Autorität von Oskar Lange (dem damaligen Botschafter der polnischen Republik in den USA) und hob die Worte des Ökonomen zu diesem Thema mit einer speziellen Schrift hervor: „Diese Grenzen [des historischen Materialismus] entstehen, weil die Ursache-Wirkungs-Beziehung, die die Theorie des historischen Materialismus formuliert, soziale Massenprozesse abdeckt, jedoch nicht das Verhalten von Individuen und nur auf einer bestimmten Ebene der sozialen Entwicklung“ (Chałasiński 1946b: 10).

Chałasiński kombinierte die Aussagen von Krzywicki und Lange und entwickelte daraus seine Idee der Anwendungsgrenzen des historischen Materialismus. Die Schriften von Lange erlaubten ihm zu argumentieren, dass „die Methode des historischen Materialismus nur eine begrenzte Anwendung in den Disziplinen haben kann, die die Bildung des individuellen menschlichen Denkens erforschen, wie zum Beispiel die Geschichte der Literatur bzw. der Philosophie“ (Chałasiński 1946b: 13). Die Texte von Krzywicki halfen ihm wiederum zu behaupten, dass „der historische Materialismus nur dann auf Disziplinen wie die Geschichte der Kunst, der Literatur oder der Philosophie angewendet werden kann, wenn es um […] Massenbewegungen geht, und nur in dem Maße, in dem die Ideen, die diese Bewegungen ausdrücken, […] den Charakter sozialer Ideen haben“ (Chałasiński 1946b: 13). Die Hervorhebung des limitierten Charakters des marxistischen Ansatzes legt dar, wie wichtig es für Chałasiński war, den Fokus der polnischen Sozialwissenschaften nicht auf den Marxismus zu reduzieren. Auf diese Weise sollten die Charakteristiken, die er seinen Protagonisten zugeschrieben hatte, gleichzeitig die Tendenzen verkörpern, die er in der neuen Realität der Nachkriegszeit für wertvoll bzw. gefährlich hielt. So schrieb Chałasiński in seinem Aufsatz: „die bemerkenswerte Stellung, die Krzywicki unter den Theoretikern des historischen Materialismus einnimmt, sollte nicht die ganzheitliche Sichtweise auf das wissenschaftliche Erbe Krzywickis einschränken, dessen Arbeiten große Bereiche der Ethnologie, Sozialgeschichte, Kulturgeschichte, Wirtschaft und Soziologie umfassten“ (Chałasiński 1946b: 14).

Die *Soziologische Rundschau* galt schnell als inoffizielles Presseorgan des polnischen soziologischen Instituts, das 1921^7^ ebenfalls von Chałasińskis Lehrer Znaniecki in Posen gegründet und nach dem Zweiten Weltkrieg von Chałasiński selbst in Łódź wiederaufgebaut wurde. Znaniecki, einer der Begründer der polnischen Soziologie, hatte Polen vor dem Krieg verlassen, um Vorlesungen an nordamerikanischen Universitäten zu halten. Nach Kriegsende hatte Znaniecki die US-Staatsbürgerschaft angenommen und wurde Professor an der Universität von Illinois in Chicago. Sein Status als Emigrant in den Westen hielt jedoch weder die Herausgeber einer vom polnischen Ministerium für Aufklärung finanzierten Zeitschrift^8^ davon ab seinen Namen zu nennen, noch verhinderte es, dass er eine Hauptrolle in der öffentlichen Debatte über die Wurzeln der polnischen Soziologie spielte.

Im Laufe der Zeit wurden im wissenschaftlichen Dialog Znanieckis Ansichten als Antithesen zu den Methoden des Marxisten Krzywicki präsentiert. Laut Chałasiński war der Ansatz von Znaniecki in der „einflussreichen intellektuellen Bewegung Ende des Jahrhunderts (das heißt Ende des 19. Jahrhunderts) verwurzelt, die für die Besonderheit (*odrębność*) der Geisteswissenschaften kämpfte“.^9^ Für Znaniecki gehörte die Soziologie fest zu den Geisteswissenschaften (*nauka humanistyczna*) und untersuchte „einen speziellen Bereich sozialer Tatsachen (*specyficzną sferę faktów społecznych*)“ (Chałasiński 1946b: 16). In seinen Werken entwickelte Znaniecki eine Vorstellung von sozialen Phänomenen und verstand diese als „eine Menge von Objekten und Handlungen“:

Objekte sind Menschen, Individuen und Gruppen als spezifische soziale Einheiten, die den Subjekten, d. h. Individuen und Gruppen, empirisch gegeben wurden; soziale Handlungen sind die Taten, die auf die Modifizierung menschlicher Individuen, auf die Veränderung oder Schaffung sozialer Gruppen zielen (Znaniecki 1922: 244).

Znaniecki verwies auf sein Konzept des „humanistischen Koeffizienten (*współczynnik humanistyczny*)“ im Forschungsprozess. Damit meinte er, dass „der Forscher sie [die kulturellen Phänomene] als jemandes bewusste Phänomene untersucht und sie so als empirische Subjekte klassifiziert, das heißt Individuen und Gruppen, die Erfahrungen haben, und sie in dem Charakter untersucht, der tatsächlich durch menschliche Erfahrungen und Handlungen erreichbar ist“ (Znaniecki 1922: 244).^10^ Chałasiński kommentierte diesen „humanistischen“ Ansatz nicht ohne Empathie, implizierte dieser doch eine „gegenseitige Kommunikation“ zwischen dem Forscher und den untersuchten Phänomenen. Znaniecki seien sowohl Historismus als auch Psychologismus fremd gewesen. Nach Chałasińskis Ansicht bemühte sich Znaniecki, bei der Förderung seiner Idee einer besonderen „kulturellen Realität“ (Znaniecki 1919)^11^ „eine Lehre (*nauka*) von sich ständig wiederholenden Elementen der sozialen Realität zu schaffen, indem er diese Elemente als sowohl vom biologischen Substrat als auch von der historischen Realität unabhängig bezeichnet hatte“ (Chałasiński 1946b: 18).

Nachdem Chałasiński das Erbe der beiden Hauptfiguren der polnischen Sozialwissenschaften der Zwischenkriegszeit – Znaniecki und Krzywicki – diskutiert hatte, machte er einige Bemerkungen zur Lage der polnischen Soziologie im Vergleich zu anderen Disziplinen. Chałasiński wollte die aktuelle wissenschaftliche Agenda beeinflussen und nutzte die polnische soziologische Tradition, um darauf zu drängen, dass die Geschichtsschreibung in Polen unter den neuen Bedingungen in Richtung der Soziologie bewegt werden musste. Zwei Hauptfiguren, die er in seinem Artikel anführte, waren der Soziologe und Rechtswissenschaftler Ludwik Gumplowicz (1838–1909), der 1893 Professor an der Universität Graz geworden war, und der prominente polnische Soziologe Stefan Czarnowski (1879–1937), der eine der ersten polnischen Professuren für „Soziologie und Kulturgeschichte“ an der Universität Warschau innegehalten hatte. Beide Wissenschaftler hatten soziologische Ansätze für die Geschichtsschreibung entwickelt und schienen in ihren Urteilen ziemlich kategorisch zu sein. Gumplowicz schrieb in dem von Chałasiński zitierten Ausschnitt: „Es steht außer Frage, dass die Geschichte der Kampf zwischen einigen sozialen Gruppen gegen andere ist […] diese Vision […] ist weder materialistisch noch idealistisch, sondern nur soziologisch“ (Chałasiński 1946b: 25). Laut Czarnowski „ist ein Historiker in der Lage, die Tatsachen des allgemeinen Prozesses zu erkennen, soweit er sie als Veränderungen von Typen klassifizieren kann […]. Er sollte die Norm kennen […]. Daher ist es eine notwendige Voraussetzung für ihn, um seine Aufgaben zu erfüllen, dass er ein Soziologe sein muss.“^12^

Auf diese Weise entwickelte Chałasiński die Idee des soziologischen Ansatzes in der Geschichtswissenschaft und bezog sich dabei auf seine eigene Forschung zur Erziehungsgeschichte. Chałasiński kombinierte Elemente von Znanieckis und Czarnowskis soziologischen Programmen und wies darauf hin, dass er in seiner Analyse mittelalterlicher Bildungspraktiken die Unterbringung von Kindern außerhalb ihres Familienhauses zwecks einer beruflichen Ausbildung als „soziale Institution“ interpretiert habe. Dieser Prozess erfordere eine spezielle Untersuchung, die ohne „die traditionelle historische Perspektive durch die soziologische Perspektive zu ersetzen“ unmöglich gewesen sei. Außerdem merkte Chałasiński an, dass viele verschiedene Gesellschaften gleichzeitig koexistieren und je eigene Entwicklungslogiken aufwiesen. Seiner Meinung nach sollte es die Aufgabe der Soziologen sein, mit soziologischen Abstraktionen zu arbeiten, ohne die Vision der Gesellschaft zu verlieren, zu der diese „sozialen Institutionen“ gehören. Chałasiński warf Historikern ihre archaischen Gesellschaftsvisionen vor und schrieb:

die alten abstrakten Konzepte der Gesellschaft [verwerfend], die für Comte und Spencer üblich waren und unter Historikern immer noch populär sind, [geht] der moderne Soziologe von der Annahme aus, dass es mehrere Gesellschaften mit unterschiedlichen Strukturen gibt; er fragt nach den eigenen Institutionen jeder Gesellschaft und repräsentiert sie in Hinsicht auf ihren Platz in der früheren Gesellschaft. (Chałasiński 1946b: 29)

Seine Skepsis gegenüber der marxistischen Theorie veranlasste Chałasiński zu einer Kritik des ökonomischen Determinismus in der Geschichtsschreibung. Er nutzte dieses Thema, um wiederholt den begrenzten Charakter der marxistischen Gesellschaftsvision hervorzuheben und somit Kritik am Marxismus selbst zu üben. So schrieb er: „Aus soziologischer Sicht ist es auch in Bezug auf die moderne Gesellschaft unvernünftig, die Sozialgeschichte als etwas zu betrachten, das der Wirtschaftsgeschichte nachgeordnet ist“ (Chałasiński 1946b: 30). Gleichzeitig stellte Chałasiński die Bedeutung wirtschaftlicher Prozesse der gesellschaftlichen Entwicklung nicht in Frage. Auf diese Weise verwies er auf seine eigenen Forschungen zu polnischen Auswanderern in den USA, die nach Chałasińskis eigener Aussage sowohl von der „amerikanischen historischen Schule“ Frederick Jackson Turners (1861–1932) als auch von dem „wirtschaftlichen Ansatz in der Geschichtsschreibung“ Charles Austin Beards (1874–1948) beeinflusst worden waren (Chałasiński 1946b: 31). So stellte er seine Vorstellungen über den Wirtschaftsfaktor in der Geschichte und den sozialen Wandel heraus und betonte, dass „der soziologische Standpunkt den wirtschaftlichen Ansatz nicht ausschließt, wenn er durch die historisch ausgeprägte Struktur der untersuchenden Gesellschaft und durch die Art der untersuchten Institutionen gerechtfertigt ist“ (Chałasiński 1946b: 30).

## Die ersten Schritte der Stalinisierung und die „Radikalisierung“ der polnischen Soziologie

Einer der aussagekräftigsten Texte, die das Ethos der polnischen Soziologie in der öffentlichen Reflexion der Nachkriegszeit illustrieren, ist Chałasińskis Beitrag „Zum dreißigjährigen Bestehen der polnischen Soziologie“. Vor dem Hintergrund der politischen Ereignisse 1948/49, als die Stalinisierung des öffentlichen Raums Polens bereits begonnen hatte, ist es aufschlussreich, wie Chałasiński die Genealogie der polnischen Sozialwissenschaften darstellte. In dem 1949 erschienenen Aufsatz verwies er darauf, dass die Wurzeln der polnisch-soziologischen Tradition in der publizistischen Kultur des späten 19. Jahrhunderts liegen. Den Ausgangspunkt der Fachgeschichte der polnischen Sozialwissenschaften betreffend bemerkte Chałasiński, dass Ludwik Gumplowicz bereits 1885 das Thema Soziologie in seinem Artikel „Der erste polnische Soziologe“ angesprochen habe.^13^ Darin setzte er sich mit den Schriften des Philosophen und Ökonomen Józef Supiński (1804–1893) auseinander, dessen Buch *Die polnische Schule der Sozialökonomie* (Supiński 1865) ihn zum Begründer des polnischen Positivismus und der Theorie der „organischen Arbeit“ machte (Polakowska & Gacowa 2003: 170–171).^14^

Der Einfluss des Positivismus von Auguste Comtes auf Supiński war aber nicht der entscheidende Punkt der von Chałasiński imaginierten soziologischen Tradition in Polen. Er betonte vielmehr, dass in der zweiten Hälfte des 19. Jahrhunderts die „Soziologie von Aktivisten und Publizisten praktiziert [wurde], für die der soziologische Journalismus das Instrument ihrer sozialen und nationalen Tätigkeit war“ (Chałasiński 1949: 2–3). Diese Idee führte Chałasiński zu der Auffassung, dass die Meinungen der polnischen Soziologen häufig von ihren ideologischen Zugehörigkeiten beeinflusst wurden. Er argumentierte in demselben Artikel weiterhin: „Die polnische Soziologie stand in engster Verbindung mit den theoretischen Ideen des Marxismus und aus sozio-ideologischer Sicht mit [dem] Sozialismus sowie mit den fortschrittlichen populären und liberalen Bewegungen“ (Chałasiński 1949: 3). An dieser Stelle war es für Chałasiński wichtiger, nicht die Grenze des Marxismus aufzuzeigen, sondern zu beweisen, dass Soziologen dem „fortschrittlichen Lager“ angehörten. Laut Chałaśiński machte das ideologische Engagement die Soziologie auch in gewissem Sinne zum Opfer der „reaktionären Vergangenheit“ Polens und verursachte wesentliche Schwierigkeiten bei der Kommunikation mit den traditionellen akademischen Disziplinen. Chałaśiński bezog sich unter anderem auf den Fall von Franciszek Bujak (1875–1953), einem prominenten polnischen Sozialhistoriker, der in konservativen Professorenkreisen unter den Verdacht geriet, sozialistische Sympathien gehegt zu haben, was sich zwangsläufig auf sein Promotionsverfahren auswirkte.

Dennoch öffneten laut Chałaśiński die akademischen Institutionen Lembergs und Warschaus – wenn auch widerwillig – ihre Türen den polnischen Sozialwissenschaften, während die Universität Krakau sich entschieden dagegen positionierte (Chałasiński 1949: 8–9). Chałasiński, einer der Initiatoren der „Volksreform“ an polnischen Universitäten,^15^ Professor und späterer Rektor der neu gegründeten „Öffentlichen Forschungsuniversität“ in Lódź (1945), arbeitete in seinem Artikel insbesondere heraus, dass neue „fortschrittliche“ Universitäten der Zwischenkriegszeit (zum Beispiel die Universität in Posen, Sitz des soziologischen Instituts von Znaniecki) viel flexibler mit der jungen Disziplin der Soziologie umgegangen seien und diese aufgenommen hätten (Chałasiński 1949: 18). Auf diese Weise wurde die „Polnische Volkshochschule“ in Warschau (*Wolna Wszechnica Polska*) mit ihrer Abteilung für Soziologie zu einer wichtigen Plattform für die Entwicklung der soziologischen Lehre. Unter anderem zogen diese Institutionen mehrere Soziologen an, die später die Agenda der polnischen Sozialwissenschaften mitbestimmten.^16^ Auf der Suche nach der Einheit der Geschichte der polnischen Soziologie betonte Chałasiński die besondere Rolle des „Instituts für soziale Fragen“ (*Instytut Spraw Społecznych*), das unter der Leitung des Pädagogen und Aktivisten Kazimierz Korniłowicz (1892–1939) gestanden hatte. Gleiche Bedeutung maß er dem 1936 gegründeten staatlichen „Institut für bäuerliche Kultur“ (*Państwowy Instytut Kultury Wsi*) bei, das von Chałasiński selbst geleitet worden war. Beide Institute wurden zu Orten der Kommunikation zwischen Soziologen unterschiedlicher ideologischer Zugehörigkeit (Chałasiński 1949: 10–11).

Chałasiński, der sich selbst als „Znanieckis Mann (*znaniecczyk*)“ charakterisierte, zögerte nicht, das Zwischenkriegskollektiv der soziologischen Zeitschrift und des Posener Instituts, dem er angehörte, als ein „liberal-demokratisches Milieu“ zu bezeichnen. Laut Chałasiński entsprach das liberale Ethos dieser Organisationen „weder der Ideologie der intellektuellen Kreise von ‚Endecja‘^17^ noch der [Ideologie] der ‚sanationischen‘^18^ Faschisten“ (Chałasiński 1949: 15). In eher kritischem Ton schrieb der Bauernsohn Chałasiński, dass die Mitglieder der Posener Gruppe von Soziologen, die von Znaniecki angeführt wurden, im Gegensatz zu Krzywiskis Männern „derselben Klasse polnischer Europäer angehörten“: Da sie alle eine „Auslandserziehung (*zagraniczne wykształcenie*)“ genossen hatten, waren sie so „Liberale französisch-angelsächsischen Typs (*liberałowie francusko-anglosaskiego pokroju*)“ ohne aktive Teilnahme an sozialen Bewegungen (Chałasiński 1949: 13–14).

Die Veränderungen in Chałasińskis Darstellung der Genealogie der polnischen Sozialwissenschaften in der „Soziologische Rundschau“ zwischen 1945 und 1949 folgen einer gewissen Logik. Unmittelbar nach Kriegsende betonte er insbesondere die Grenzen der Anwendung des Marxismus. Seiner Vorstellung nach sollte die polnische Soziologie die Vielfalt des Zwischenkriegserbes entwickeln und sich nicht lediglich auf den marxistischen Ansatz beschränken. In späteren Publikationen, die schon in die erste Phase der Stalinisierung fielen, schien es eher notwendig, die Fortschrittlichkeit der Soziologen zu betonen. In einer Anekdote, mit der Chałasiński seine Ode an die „wiederbelebte Soziologie“ schloss, erinnerte er sich an ein Gespräch zwischen ihm und dem Soziologen Stefan Czarnowski im Jahr 1934. Das Schicksal der soziologischen Zeitschrift betreffend sagte Czarnowski skeptisch: „Die Veröffentlichung einer soziologischen Zeitschrift kann in Polen nicht erfolgreich sein, wenn sie in Frankreich fehlschlug, wo *L’année sociologique* nicht mehr veröffentlicht wird“ (Chałasiński 1949: 54). Rund 15 Jahre später antwortete Chałasiński seinem Opponenten: „Es hat in Polen doch geklappt. Wir feiern jetzt das Jubiläum des Vertrauens in die polnischen Möglichkeiten und Kräfte“ (Chałasiński 1949: 54). Ironischerweise erschien diese Anekdote in der letzten Ausgabe der Zeitschrift vor ihrer Einstellung in Folge neuer Maßnahmen der „Stalinisierung“ des polnischen akademischen Lebens.

## Auf dem Weg zur Einheit: Die „fortschrittliche“ Tradition als politische Aufgabe

Die politischen Veränderungen zwischen 1949 und 1951 transformierten den akademischen Status der Soziologie radikal. Die Reorganisation der Abteilung für Soziologie an der Universität Warschau zwischen 1949 und 1950 (Sułek 2020: 314–317), die Unterbrechung der Aktivitäten des polnischen Instituts für Soziologie in Łódź (1951) sowie die Schließung der *Soziologischen Rundschau* (1949) waren eindeutige Anzeichen dafür, dass eine Diskussion über die Genealogie der polnischen Sozialwissenschaften in ihrer früheren Form nicht mehr möglich war. Der strukturelle Wandel in den Propaganda- und Zensurinstitutionen sowie die Neuausrichtung der Kultur- und Wissenschaftszeitschriften, die auf eine propagandistische Art der Berichterstattung über Kultur- und Wissenschaftsereignisse getrimmt wurden, führten dazu, dass die Kultur- und Wissenschaftspresse ihre Rolle als Plattform für soziologische Debatten verlor. Interessanterweise blieben im akademischen Diskurs die Begriffe „Soziologie“ und „Soziologe“ erhalten, während sie aus den Namen der Abteilungen verschwanden. Das Projekt des „Ersten Kongresses der polnischen Wissenschaft“, dessen Planung lange vor der feierlichen Veranstaltung im Sommer 1951 (Hübner 1983: 73–87) begonnen hatte, sollte dazu beitragen, das akademische Leben Polens „von oben“ zu organisieren. Während es unmittelbar nach dem Krieg in der Kultur- und Wissenschaftspolitik zum Mainstream wurde, die komplexe und vielfältige polnische Wissenschaftslandschaft an die allgemeinen Postulate der neuen Realität anzupassen, führten die politischen Veränderungen im polnischen Staat während der Stalinisierung zu einer Dominanz der Mobilisierungsrhethorik in der Wissenschaft.

Die staatlichen Maßnahmen im Zuge der Neuorganisation unterschiedlicher Disziplinen hatten zur Folge, dass sich beim Kongress „Philosophie und Soziologie“ eine Untersektion teilen mussten.^19^ Diese Zusammenführung verursachte Komplikationen, da vielfältige, häufig miteinander im Widerspruch stehende wissenschaftliche Ansätze auf einen gemeinsamen Nenner gebracht werden mussten. Die „Gleichschaltung“ des öffentlichen Raums wurde also zu keinem Hindernis um sowohl offiziell anerkannte Sozialwissenschaftler, die ihre Lehren an den Universitäten unter den neuen Bedingungen fortgesetzt hatten,^20^ als auch die Akademiker, die in der Zeit der Stalinisierung vom Unterricht suspendiert worden waren,^21^ zur Teilnahme an den Diskussionen einzuladen. Darüber hinaus wurde Chałasiński, der sich nie als Marxist bezeichnet hatte, zum Vorsitzenden der Sektion für Sozial- und Geisteswissenschaften ernannt.^22^ Zudem besetzte er die führende Position im Exekutivkomitee des Kongresses. Es ist bemerkenswert, dass die Untersektion für Philosophie und Sozialwissenschaften von Kazimierz Ajdukiewicz (1890–1963), einem der prominentesten Vertreter der Lemberg-Warschau-Schule geleitet wurde,^23^ der kaum als Sympathisant des Marxismus-Leninismus bezeichnet werden kann. Die Listen der Teilnehmer an den Vorbereitungsveranstaltungen des Kongresses^24^ zeigen sehr deutlich, dass nicht ideologische Auffassung, sondern Zugehörigkeit zur „akademischen Gemeinschaft“ als Kriterium für die Rekrutierung diente.^25^

Die in der Presse proklamierte und propagierte allgemeine „wissenschaftliche Einheit“^26^ entsprach jedoch kaum dem Inhalt und der Logik der Debatten bei den Vorbereitungssitzungen. Kazimierz Ajdukiewicz,^27^ Philosoph und Schüler des bekannten polnischen Logikers Kazimierz Twardowski (1866–1938), berichtete in einem seiner Vorträge über die Arbeit der Untersektion für Philosophie und Sozialwissenschaften, dass „entschieden wurde, die Referenten, die meistens keine Marxisten sind, nicht zu zwingen, ihre Kritik aus marxistischer Sicht auszuüben, sondern zu erlauben, die Kritik aus ihrer eigenen Sicht zu leisten.“^28^ Der marxistische Soziologe Julian Hochfeld (1911–1966) argumentierte hingegen, dass der Kongress „nicht die Summe der Kongresse einzelner Disziplinen sein sollte. Der Kongress der polnischen Wissenschaft sollte ein Akt der Einheit der Wissenschaft werden.“^29^ Dennoch wollte auch Hochfeld nicht alle dazu zwingen, dem marxistischen Ansatz zu folgen. Hochfeld argumentierte sogar, dass er nicht verstehe, wie es möglich sei, Themen „aus marxistischer Sicht“ oder „aus ihrer eigenen Sicht“ zu betrachten, wo es doch die Pflicht „jedes verantwortungsbewussten Gelehrten“ sei, alle Fragen aus den eigenen Überzeugungen heraus zu beantworten. Außerdem betonte Hochfeld: „Wenn jemand nicht von der Gerechtigkeit dieser [marxistischen] Sichtweise der Welt überzeugt [ist], so ist es sein Recht und sogar seine Pflicht, nicht die marxistische Auffassung zu unterstützen, sondern klar zu beweisen, dass er [als Opponent des Marxismus] Recht hat.“^30^

In weiteren rhetorischen Wendungen behauptete Hochfeld auf sehr emotionale Art und Weise, dass das Fehlen einer Bereitschaft, sich in der Diskussion mit unterschiedlichen Ansätzen auseinanderzusetzen, zu einer „Art von Stagnation (*pewny marazm*)“ geführt habe, die man in der Arbeit der geisteswissenschaftlichen Sektion im Allgemeinen und der Untersektion für Philosophie und Sozialwissenschaften im Besonderen beobachten könne.^31^ Hochfeld bestand darauf, konkrete Aufgaben und Fragen für die Diskussionen zu formulieren und in der Debatte die Einheit zu demonstrieren. Als Beispiel für eine solche Frage verwies er auf die Möglichkeit, die „fortschrittliche Tradition“ der polnischen Soziologie durch Auseinandersetzung mit den Schriften ihrer besten Vertreter zu bestimmen: „Fortschrittliche soziologische Gruppen spielten eine außergewöhnliche Rolle und kritisierten die Kanons der Wissenschaftler, die vollkommen im Idealismus versunken waren“. Für Hochfeld war klar: „die progressive Rolle von zumindest einem Teil der polnischen Soziologen wie Krzywicki, Chałasiński oder Ossowski“ liegt darin, dass sie „die soziale Bedingtheit von […] anderen Disziplinen gezeigt hatten“ und damit einen Schritt in Richtung des wissenschaftlichen Fortschritts getan hätten.^32^ Unter Bezugnahme auf Friedrich Engels betonte er jedoch, dass die Zeit gekommen sei, die Sprachen der verschiedenen Disziplinen zu vereinen, „in einer einfachen Sprache zu sprechen, die ein einfacher Mann spricht“ und gleichzeitig „nicht zu vermeiden, eine politische Sprache zu benutzen, weil es sich um ein politisches Thema handelt.“^33^

Ein weiterer wichtiger Aspekt, der im Zusammenhang mit den Vorbereitungen für den Kongress hervorgehoben werden muss, ist die Teilnahme von Politikern an den Vorbereitungssitzungen. Unter den Rednern, die an den Diskussionen teilnahmen, befanden sich mehrere, deren Status besondere Aufmerksamkeit verdient. Stanisław Leszczycki, Organisator der Polnischen Geographischen Gesellschaft, Sejm-Abgeordneter und Mitglied des Hauptrats für Wissenschaft und Hochschulbildung (*Rada Główna Nauki i Szkolnictwa Wyższego*), kritisierte während seiner Teilnahme an einer der Vorbereitungssitzungen die Trägheit der Wissenschaftler bei der Erfüllung der Aufgaben des Kongresses. Als Beispiel nahm Leszczycki die Naturwissenschaften und sagte: „Sind die Geisteswissenschaftler in Polen mehr überarbeitet als die Naturwissenschaftler?“ Der Geograph und Politiker, der Meinungsverschiedenheiten als Disziplinlosigkeit betrachtete, formulierte seine Kritik folgendermaßen: „die Zahl der Geisteswissenschaftler ist natürlich größer als die der Mathematiker […] aber die Zahl kann in dieser Frage keine Rolle spielen. Ich denke, dass die Gründe [für die Trägheit der Geisteswissenschaftler] in der Ideologie gesucht werden sollten“, und fügte noch hinzu, dass seiner Auffassung nach „alle Schwierigkeiten in der Mentalität der Wissenschaftlichen Mitarbeiter“ liegen würden.^34^ Andererseits betonte Leszczycki, dass, „nur Wissenschaftler Wissenschaft machen“ sollten: Das Ziel des Kongresses sei, die „Wissenschaftler über diese Themen diskutieren zu lassen“. Leszczycki behauptete weiterhin, dass „wenn diese [die akademische Diskussion] geleitet würde, hätte es keinen Sinn Konferenzen zu machen, man könnte einfach Zirkulare schreiben“; es sei „nicht der Zweck der Untersektionen, Berichte für die Behörden zu schreiben, sondern der Zweck [sei], die Fehler durch gegenseitige Kritik herauszufinden“.^35^

Auch die Politikerin Eugenia Krassowska-Jodłowska, ihrerseits Philologin, Mitglied der Demokratischen Partei (*Stronnictwo Demokratyczne*) und Staatssekretärin (*sekretarz stanu*) im Ministerium für Wissenschaft und Hochschulwesen, beteiligte sich an der Vorbereitung des Kongresses. Auch sie kritisierte die Passivität der Sektionsmitglieder bei der Such nach einem einheitlichen Ansatz für die Sozial- und Geisteswissenschaften. Ähnlich wie Hochfeld diagnostizierte auch sie „einige Symptome von Stagnation“^36^ in der Aktivität der „geisteswissenschaftlichen“ Sektion und betonte, dass die Meinungsverschiedenheit die Einheitslosigkeit nicht entschuldige. Mit der Aussage, „man sagt über einen Aufsatz, dass er nicht marxistisch ist, aber es muss gesagt werden, warum der nicht marxistisch ist. Das Ziel ist zu zeigen, welchen wissenschaftlichen Wert der Aufsatz hat, der bürgerliche Konzepte behandelt, und zu welchen wissenschaftlichen Ergebnissen dieser führt,“^37^ kritisierte Krassowska die Teilnehmer. Sie betonte ferner, dass „niemand vorschlägt, Regeln bzw. Vorschriften durchzusetzen und Wissenschaftliche Mitarbeiter dazu zu zwingen, sie einzuhalten. Das führt zu nichts. Die Wissenschaft wird selbst ihre wissenschaftlichen Normen im Kampf, in der Auseinandersetzung zwischen verschiedenen Ansichten, entwickeln.“^38^

## Verschärfung des politischen Anspruchs, mangelnder Konsens und Verzicht auf eine Genealogie der polnischen Sozialwissenschaften

Die erhaltenen Sitzungsprotokolle der letzten Vorbereitungstreffen für den Kongress dokumentieren die von den Mitgliedern der Untersektion für Philosophie und Sozialwissenschaften geführten Debatte über „fortschrittliche Tradition“. Die von den Politiker*innen geforderte Einheit oder ein Kompromiss wurden nicht gefunden. Vielmehr konnte beobachtet werden, wie sich die politische Landschaft in Polen veränderte. Darüber hinaus muss hervorgehoben werden, dass die Schlussdiskussion über die „fortschrittliche Tradition“ der polnischen Sozial- und Geisteswissenschaften im Januar 1951 stattfand, als die führenden Mitglieder der Sektion schon davon ausgingen, dass die „Ergebnisse“ der Sektionsaktivität in wenigen Monaten in Anwesenheit von internationalem Publikum von Akademikern und Politikern präsentiert werden mussten. In Abwesenheit von Kazimierz Ajdukiewicz wurde das Treffen vom Soziologen Stanisław Ossowski (1897–1963) moderiert. Dieser begann seinen Vortrag mit einer Bemerkung über den offiziellen Bericht, der die Ergebnisse der Arbeit der Untersektion zusammenfasste und noch einmal einen Mangel an Einigung thematisierte.

Ein Vortrag des prominenten Logikers und Ethikers Tadeusz Kotarbiński (1886–1981) machte die Situation noch komplizierter. In seiner Rede schlug Kotarbiński vor, Fragen der Weltanschauung zu ignorieren und sich auf praktische Fragen zu konzentrieren. Als einer der Hauptopponenten des Marxismus hielt es Kotarbiński für notwendig, „die Differenzierung in den Forschungsbereichen und bis zu einem gewissen Grad auch die Gleichberechtigung der verschiedenen Ansätze“ zu tolerieren. In Bezug auf die Vergangenheit wies Kotarbiński auf die „passive Einstellung“ der polnischen Wissenschaftler zum Marxismus sowie auf den starken Einfluss der „Warschauer Schule der Logik“ sowohl in Polen als auch auf internationaler Ebene hin. Kotarbiński beschrieb die „aktuelle Situation“ der polnischen Philosophie und Sozialwissenschaften auf folgende Weise:

1) die soziale und parteipolitische Nachfrage der Philosophie des Marxismus-Leninismus im Kontext des Verständnisses der Philosophie als Instrument des politischen Kampfes; 2) das Postulat sich in der akademischen Arbeit ein Beispiel an den Philosophen der Sowjetunion zu nehmen; 3) […] unmaskierte Unterstützung für [die] einzige philosophische Richtung: Marxismus-Leninismus; 4) […] Neuorganisierung der Lehrstühle; Kollektivierung der akademischen Forschung; Abhängigkeit vom gesamtstaatlichen Sechsjahresplan.^39^

Kotarbiński sprach in seinem Vortrag unter anderem auch über die von ihm wahrgenommenen Errungenschaften der Zwischenkriegszeit und erwähnte dabei „den Bruch mit den Relikten des theologisch-mittelalterlichen Denkens“ und die „Disziplinierung“ des philosophischen Denkens. Mit diesen Argumenten betonte Kotarbiński, dass die so typische für das intellektuelle Leben in der Zwischenkriegszeit „freie Diskussion zwischen den Vertretern unterschiedlicher Ansätze“, „der beste Weg sei, zu korrekten Schlussfolgerungen zu gelangen.“^40^ Darüber hinaus hob er hervor, dass es „sehr wünschenswert und nützlich für beide Seiten wäre, den Meinungsaustausch zwischen polnischen und sowjetischen Philosophen zu stärken. Das Verhältnis sollte hier jedoch [vielmehr] als eine Partnerschaft auf Augenhöhe gesehen werden, nicht als einseitige Nachahmung.“^41^

Eine Antwort auf Kotarbińskis Rede kam von Józef Chałasiński, dessen Reaktion als ein anschauliches Beispiel für die Veränderungen im politischen Kontext seit 1948 dienen kann. Nach Erhalt der Sitzungsprotokolle ergriff Chałasiński während der Auseinandersetzungen auf den Vorbereitungssitzungen trotz seiner Führungsposition recht widerwillig das Wort und beschränkte sich in der Regel auf das Moderieren der Diskussion zwischen den Wissenschaftlern. Umso wertvoller sind Hinweise auf seine Aussagen während der Sitzungen. Laut der Sitzungsprotokolle kritisierte Chałasiński, der zu dem Zeitpunkt Rektor der Universität Łódź war, die Aussagen von Kotarbiński aufs Äußerste. Dabei ist besonders auffällig, dass sich Chałasiński binnen weniger Jahre eines gänzlich anderen Vokabulars bediente: In einer emotionalen Rede bezeichnete Chałasiński den Ansatz von Kotarbiński als „nicht-historisch“. Außerdem sei „die Toleranz der Regierung gegenüber der philosophischen Forschung [in der Zwischenkriegszeit] […] durch die klassenpolitischen Bedingungen des Sozialsystems bestimmt“ worden, zumal die Behörden die Tendenzen der „Isolation von der historischen Realität“, die von philosophischen Kreisen praktiziert wurden, unterstützt hätten.^42^

Mit seiner Kritik wollte Chałasiński die Notwendigkeit unterstreichen, beim Nachdenken über die Genealogie der polnischen Sozial- und Geisteswissenschaften die neue historische Realität des polnischen Staates zu berücksichtigen: „Vor dem Krieg waren wir im Zeitalter des Kapitalismus versumpft und waren die Peripherie der Welt, während wir heute im Bündnis mit der Sowjetunion, ihre Avantgarde sind.“^43^ Darüber hinaus fällte Chałasiński mehrere andere auffällige Urteile. Seiner Aussage nach waren „es nur methodisch richtige und gründlich durchdachte Thesen, die aus Diagnosen der aktuellen sozialen Entwicklung und generell des gegenwärtigen Momentes stammen, aus solchen Diagnosen, die auf der einzigen wissenschaftlichen Methode beruhen – auf dem Marxismus“.^44^ Im weiteren Verlauf seines Vortrags betonte er zudem, dass „die Umsetzung des Sechsjahresplans als die Grundlage des neuen Systems eine Frage von weitaus größerer Bedeutung ist als jede Form von geistiger Freiheit.“^45^

Auch der marxistische Philosoph Adam Schaff (1913–2006) nahm in seinem Vortrag eine Gegenposition zu Kotarbiński ein:^46^ „Niemand, der vernünftig ist, postuliert, dass die polnischen Philosophen einseitig die sowjetischen nachahmen müssen.“^47^ Dennoch stimmte Schaff Chałasiński zu und charakterisierte Kotarbińskis Ansatz der wissenschaftlichen Freiheit als weder „historisch“ noch „soziologisch“. Seiner Ansicht nach könne jemand, der Kotarbińskis Rede höre, „die völlig falsche Schlussfolgerung ziehen, dass die Situation vor dem Krieg besser als die derzeitige“ sei.^48^ Darüber hinaus spitzte Schaff das Ganze mit den Worten zu:

Die von Professor Kotarbinski eingeführte Postulierung der Diskussionsfreiheit ist ein weiteres Ergebnis seines nicht soziologischen, abstrakten Ansatzes. Sie könnte erst dann akzeptiert werden, wenn es keine fremden Geheimdienste des Klassenfeindes, keine reaktionären Banden mehr gäbe, und wenn es tatsächlich die nur abstrakte Situation einer gleichen Anzahl bürgerlicher und marxistischer Wissenschaftler gäbe.^49^

Schaffs Begründung der numerischen Ungleichheit von marxistischen und nichtmarxistischen Wissenschaftlern ist äußerst interessant:

Der Klassenkampf geht weiter, und im wissenschaftlichen Bereich haben wir, statt einer gleichen Anzahl von Disputanten einerseits das große Lager der Phänomenologen, Neothomisten usw., andererseits das marxistische Lager, das in seiner Perspektive zwar groß, zahlenmäßig aber tatsächlich immer noch klein ist – freie Diskussion wäre [in dieser Situation] die größte Nachlässigkeit, mehr noch, – ein soziales Verbrechen.^50^

Die festgefahrene Situation innerhalb der Diskussion lässt sich anhand der Strategie von Stanisław Ossowski zur Verteidigung der Meinung seines Lehrers Kotarbiński veranschaulichen. Er wiederholte die große Bedeutung der Mobilisierung aller Wissenschaftler, um zur Umsetzung des Sechsjahresplans beizutragen, fügte aber hinzu, dass „die Mobilisierung von intellektuellen Kräften ohne die Freiheit der Diskussion unmöglich ist.“ Laut Ossowski könne die Verletzung dieser Freiheit zu einer Stagnation der intellektuellen Entwicklung führen, weil „einer, der einen Apfelbaum fällt, um schneller Äpfel zu pflücken, nicht den Regeln der rationalen Produktion folgt“.^51^ In seiner Reaktion auf diese Aussage meinte Chałasiński, dass die Diskussionsfreiheit „in einem engen Kreis von Professoren“, aber nicht „in weiten Kreisen von Studenten“ zugelassen werden sollte, da dies ein Faktor für die „Demobilisierung der sozialen Jugendkräfte“ werden könnte.^52^

Aus den Debatten geht hervor, dass die Teilnehmer, die „eine fortschrittliche Tradition der polnischen Sozialwissenschaften“ herausarbeiten sollten, es in der Vorbereitungszeit nicht geschafft haben, die grundlegenden Widersprüche in ihren Ansätzen zu lösen. Angesichts der komplexen Situation versuchte Ossowski, einen Kompromiss zwischen der neuen Realität und dem intellektuellen Erbe der Zwischenkriegszeit zu finden. Seiner Ansicht nach bezeugten die Vorträge der Sektionsmitglieder, dass in intellektuellen Kreisen der Zwischenkriegszeit kein wesentliches Interesse am Marxismus bestanden habe. Ossowski betonte außerdem, dass der „Kult der wissenschaftlichen Genauigkeit“ (*kult ścisłości naukowej*) ein sehr wichtiger Verdienst der Geisteswissenschaften der Zwischenkriegszeit sei, gerade vor dem Hintergrund der Dominanz der mittelalterlich-theologischen Herangehensweise in der Philosophie.

Dem Archivmaterial zufolge hatten die Diskussionsteilnehmer zur letzten Vorbereitungssitzung einen Entwurf des Sektionsberichtes erstellt, der angeblich vom Soziologen Tadeusz Szczurkiewicz (1895–1984) vorbereitet wurde.^53^ Da dieser recht triviale Entwurf als Grundlage des offiziellen Berichts genommen wurde, ist es wichtig die Reaktion der Diskutanten auf die Hauptthesen des Berichtes näher zu betrachten. Der charakteristischste Beitrag betraf Florian Znaniecki, der im Bericht als „Idealist“ bezeichnet wurde: Während laut Sitzungsprotokollen „Znanieckis Mann“, sprich: Chałasiński, an dieser Stelle geschwiegen haben soll, versuchte Ossowski den Vorwurf des Idealismus gegen Znaniecki zurückzuweisen. Auf der Suche nach einem Interessenausgleich vertrat Ossowski die Auffassung, dass Znanieckis „humanistischer Koeffizient“ ein sehr komplexes Konzept sei, das nichts mit Idealismus zu tun habe. Darüber hinaus gebe es „unter den ernsthaften modernen bürgerlichen Soziologen niemanden, der die Grundprinzipien des historischen Materialismus nicht anerkennen würde.“^54^ Dieser Versuch, alle intellektuellen Richtungen als tatsächlich marxistische darzustellen, rief einen starken Protest von Seiten Szczurkiewiczs und Schaffs hervor, die Ossowski beschuldigten, Znanieckis Konzept falsch verstanden zu haben und den Unterschied zwischen marxistischen und bürgerlichen Wissenschaften zu verwischen. Ossowskis Kommentar, er sähe „keine Möglichkeit […], den Streit über die Absicht von Znaniecki zu lösen, weil seine Konzepte nicht präzise genug waren“, was „ein Beispiel einer häufigen Krankheit der Sozialwissenschaften“^55^ sei, illustriert anschaulich den „Kompromiss“ der Mitglieder der Untersektion für Sozial- und Geisteswissenschaften kurz vor dem zeremoniellen Teil des Kongresses.

Es ist auffällig, dass die von den Sektionsmitgliedern geführten Debatten kaum inhaltlich die „fortschriftliche Tradition“ der polnischen Sozialwissenschaften darlegten. Schließlich hatten die Mitglieder der Untersektion die Aufgabe, sich mit der Vergangenheit aus einer zukünftigen Perspektive auseinanderzusetzen. So konnten die den unterschiedlichen Traditionen angehörenden Wissenschaftler nicht die wesentlichen Widersprüche in ihrem Verständnis der Fortschrittlichkeit lösen. Demnach hatte der offizielle Bericht der Untersektion weniger mit dem Inhalt der Debatten zu tun, die während der Vorbereitungssitzungen geführt wurden. Eine Beschreibung des Idealbildes der „sozialistischen Philosophen und Soziologen“ führte immerhin zu der Einsicht, dass die polnischen Sozialwissenschaften weiterhin unter dem starken Einfluss von „nicht soziologischen“ und „nicht historischen“ Ansätzen standen. Ebenso auffällig ist, dass die Arbeiten der aktiven Diskussionsteilnehmer Ajdukiewicz und Kotarbiński als „reaktionärer und idealistischer Konventionalismus“ bezeichnet wurden, obwohl die Leistungen der Warschauer Logikschule auf dem Gebiet der Mathematik als ein fortschrittliches Element dieser philosophischen Bewegung anerkannt wurden.^56^

Auch wurde die polnische soziologische Tradition mit einer durchaus harten Begrifflichkeit beschrieben. Laut dem offiziellen Bericht hatten die „kosmopolitischen“, „imperialistischen“ und „kapitalistischen“ Tendenzen des deutschen Idealismus sowie die neukantianischen Ansätze die polnischen Sozialwissenschaften im Allgemeinen und die Schüler von Florian Znaniecki im Besonderen tief beeinflusst. Die Werke von Ossowski, der mittlerweile stellvertretender Vorsitzender der Untersektion war, wurden als „falsch“ bezeichnet, da sie gegen die Idee der historischen Gesetze und folglich gegen den Marxismus gerichtet waren. Gleichwohl wurde betont, dass Ossowski trotz seiner liberalen, nicht sozialistischen Haltung, zum ideologischen Kampf gegen die faschistischen Denker beigetragen hätte.^57^ Selbst der marxistische Soziologe Ludwik Krzywicki erschien im Bericht nicht „sündenlos“. Bei der Entwicklung des progressiven materialistischen Programms hatte Krzywicki die Bolschewiki kritisiert. Seine Kritik wurde eindeutig als „Verleumdung der Oktoberrevolution und ihrer Führer Lenin und Stalin“ aufgefasst. Zwar wurde das soziologische Erbe von Znaniecki als „ausgesprochen rückständig (*wyraźnie wsteczny*)“ dargestellt, doch stelle man zugleich fest, dass sein Schüler Chałasiński „von Beginn seiner akademischen Tätigkeit [an] dem Wirtschaftsfaktor eine höhere Aufmerksamkeit“ in sozialen Prozessen schenkte – wohl eine Entschuldigung für das Verhältnis zu seinem „reaktionären Lehrer“. Trotzdem konnte der Sektionsvorsitzende Chałasiński dieser „offiziellen Kritik“ nicht entkommen, da einige seiner Zwischenkriegspublikationen „immer noch auf der Unterstützung der kapitalistischen Basis“ des reaktionären Zwischenkriegsregimes beruhten. 58Die polnischen Sozialwissenschaften wurden in der festlichen Atmosphäre des ersten wissenschaftlichen Kongresses also vor allem durch ihre reaktionäre Vergangenheit, problematische Gegenwart und kaum erreichbare marxistisch-leninistische Zukunft charakterisiert.

## Fazit

Das von Chałasiński direkt nach dem Krieg konstruierte Bild der polnischen Soziologie konnte kaum mit dem späteren Bild der „fortschrittlichen Tradition“ polnischer Sozialwissenschaften in Einklang gebracht werden. Chałasiński, der die *Soziologische Rundschau* wiederbelebt hatte, sah sich selbst als Fortführer der akademischen soziologischen Tradition im Nachkriegspolen. Die erste Version der Genealogie der Sozialwissenschaften, die von Chałasiński gleich nach dem Krieg verfasst wurde, sollte einerseits die Vielfalt und Aktualität der soziologischen Ansätze der Zwischenkriegszeit demonstrieren, andererseits anschaulich zeigen, dass die akademische Agenda Polens nicht auf den Marxismus reduziert werden sollte. Die Hauptfiguren der polnischen Soziologie der Zwischenkriegszeit und nicht zuletzt Marxisten hatten für Chałasiński die Grenzen der Anwendbarkeit des historischen Materialismus definiert und damit einen breiten Raum für die Entwicklung der Soziologie im Nachkriegspolen geschaffen.

Der Beginn der Stalinisierung wurde dann zum Anlass, die Perspektive auf die soziologische Tradition zu revidieren. An dieser Stelle war es wichtiger, Soziologie als fortschrittliche gesellschaftliche Bewegung darzustellen. Die Fortschrittlichkeit der soziologischen Gruppen sollte als ein Schutzargument dienen und gleichzeitig einen neuen Raum für die Soziologie als Kämpferin gegen reaktionäre Ansichten schaffen. Dennoch brachte die Stalinisierung neue Herausforderung für die Soziologen mit sich. Im Rahmen des Wissenschaftskongresses sollten die Soziologen eine „fortschriftliche“ Tradition ihrer Wissenschaft erschaffen, die aufgrund ihrer reaktionären Vergangenheit dabei war, aus dem akademischem System Polens zu verschwinden. Der Kongress wurde für die polnischen Wissenschaftler zur Aufgabe, eine „mathematische Gleichung“ mit bekanntem Ergebnis und mehreren obligatorischen Elementen wie der „Mobilisierung der besten Wissenschaftler“, der Schaffung einer „fortschrittlichen nationalen Tradition“, dem „Marxismus-Leninismus“ und dem „Sechsjahresplan“ zu schreiben.

Dennoch war die Tradition der polnischen Sozialwissenschaften stark genug, um nicht in den Sechsjahresplan gedrängt bzw. darauf reduziert zu werden. Die neue politische Realität zur Hochzeit der Stalinisierung forderte den Verzicht auf die ganze Tradition der Sozialwissenschaften. Die Soziologie aber ließ sich nicht mehr an die neuen Grenzen des Erlaubten anpassen. Die Veränderungen im öffentlichen Diskurs, die an Józef Chałasińskis Äußerungen abzulesen sind, veranschaulichen die Natur der politischen Veränderungen in Polen sowie ihren Einfluss auf die wissenschaftliche und akademische Landschaft. Chałasiński, der alle soziologischen Institutionen verloren hatte, die er nach dem Krieg gegründet hatte, wurde dennoch ordentliches Mitglied der Akademie der Wissenschaften und Rektor der neugegründeten Universität von Łódź. Es ist nicht ohne Ironie, dass die für das Regime positiv konnotierten Wörter „Soziologie“ und „Soziologe“ in den offiziellen Berichten des Kongresses bestehen blieben, während die Herkunft der Disziplin getilgt wurde.

Im Rahmen der Entstalinisierung Polens, nahm Józef Chałasiński wenige Jahre später die Neugründung seiner Zeitschrift *Soziologische Rundschau* zum Anlass, Florian Znaniecki einen Artikel zum Geburtstag zu widmen. Die Werke seines Lehrers definierte Chałasiński als einen „großen Beitrag zur soziologischen Theorie sowohl in Amerika als auch in Europa“, und bezeichnete die Veröffentlichung von Znanieckis Büchern als „den Moment des Eintritts der polnischen Soziologie in das moderne soziologische Denken“ (Chałasiński 1957: 10). Über die gesamte Periode der Volksdemokratie in Polen war die öffentliche Repräsentation der polnischen soziologischen Tradition ein wichtiger Indikator für die jeweilige Position der Soziologie in der akademischen Landschaft. Unter Berücksichtigung der offizinalen Dominanz des Marxismus-Leninismus sollten polnische Soziologen noch oft die Grenzen des Erlaubten in den öffentlichen Debatten über die wissenschaftliche Identität ihrer Disziplin ausreizen.

## Danksagung

Ich bedanke mich sehr herzlich bei Jonas Löffler, Maciej Górny, Christian Fleck, Marcin Wolniewicz, Friedrich Cain, Jan Surman und Catherine Gousseff für ihre Anmerkungen zum Manuskript dieses Aufsatzes. Dieser Aufsatz wurde im Rahmen des Projektes „European Union’s Horizon 2020 Marie Skłodowska-Curie grant agreement No 713600 (artes EUmanities)“ vorbereitet.

## Footnotes

1 Die Literatur zu diesem Thema ist umfangreich, siehe zum Beispiel Publikationen über den „Beitrag“ der Wissenschaftler zum „Krieg der Geister“ am Anfang des 20. Jahrhunderts (Górny 2019), die Teilnahme der Geografen an der Formierung der politischen Realität (Górny 2020), und die Rolle der Statistiker in der Nationsbildung (Labbé 2019). Über die Aktivität der polnischen Wissenschaftler im Kontext der nationalsozialistischen Besatzung, siehe: (Cain 2020).

2 Siehe den Programmartikel bezüglich der „sanften Revolution“ von Jerzy Borejsza, (Borejsza 1945: 1).

4 I Kongres Nauki Polskiej, Warszawa: Państwowe Wydaw. Naukowe, (1953). S. 19.

5 I Kongres Nauki Polskiej, Warszawa: Państwowe Wydaw. Naukowe, (1953). S. 110.

6 Krzywicki war eine der Hauptfiguren der Sozialwissenschaften der Zwischenkriegszeit (Szacki 2002: 502–504).

7 Das Institut wurde als „Soziologisches Institut“ gegründet, 1927 wurde es als „Polnisches Soziologisches Institut“ ernannt (Chałasiński 1949: 6–7).

8 Siehe, Przegląd Sociologyczny 1946–1947. Dennoch erscheint Znanieckis Name nicht unter den Redakteuren der Ausgabe von 1948, die 1949 erschien.

9 Es handelt sich um Wilhelm Windelband und deutsche Neukantianische Philosophen, die Znanieckis Konzept sehr deutlich beeinflusst hatten.

10 Siehe zum Konzept des „humanistischen Koeffizienten“, Hałas (2010).

11 Siehe Znaniecki (1919).

12 Chałasiński zitiert aus dem Buch (Czarnowski 1939: 26–29).

13 Der Aufsatz wurde in der polnischen Zeitschrift *Prawda (Die Wahrheit*) veröffentlicht, die von dem prominenten Schriftsteller und Publizist Aleksander Swiętochowski gegründet und herausgegeben wurde (1849–1938), Siehe: Gumplowicz Ludwik. (1885). Pierwszy socjolog polski. *Prawda* (12): 134–135; (13): 146–148; (14): 161–162; (15): 173–174.

14 Chałasiński erwähnte ks. Fr. Krupiński und seinen Aufsatz *The Positivist school (Szkoła pozytiwistyczna)*, der in der Zeitschrift *Biblioteka Warszawska (Warschauer Bibliothek*) 1868 erschien, als ein wichtiges Beispiel der Diskussion über Positivismus (Chałasiński 1949: 2)

15 Siehe zu diesem Programm, Zysiak (2016).

16 Unter anderen, haben Ludwik Krzywicki und Józef Chałasiński an dieser informellen Volkshochschule ihre Vorlesungen gehalten (Chałasiński 1948: 9).

17 Von ND – Nationaldemokratische Bewegung.

18 Von „Sanation“ – dem Regime von Józef Piłsudski.

19 Eine der Hauptaufgaben der „humanistischen“ Sektion des Kongresses war die „Popularisierung und Verherrlichung der progressiven Wissenschaftler“, siehe: Archiwum PAN, I Kongres Nauki Polskiej, Sygn. 13, K. 24.

20 E.g. Julian Hochfeld and Nina Assorodobraj-Kula, die marxistischen Ansätze zur Analyse der gesellschaftlichen Prozesse an der Universität Warschau weiter unterrichtet hatten.

21 Etwa Stanisław and Maria Ossowski.

22 Chałasiński war schon Rektor der Universität Łódź und leitete manchmal den Vorbereitungsprozess durch Telegramme (Archiwum PAN, I Kongres Nauki Polskiej, Sygn. 13, K. 46.) Es ist bemerkenswert, dass Jerzy Jedlicki, später berühmter Ideenhistoriker bei dem Kongress als wissenschaftliche Hilfskraft arbeitete (siehe: Archiwum PAN, I Kongres Nauki Polskiej, Sygn. 13, K. 7).

23 Archiwum PAN, I Kongres Nauki Polskiej, Sygn. 13, K. 115.

24 Siehe die Liste der Teilnehmer: Archiwum PAN, I Kongres Nauki Polskiej, Sygn. 13, K. 110–115.; Die spezielle Sektion für Geschichte der Aufklärung (Archiwum PAN, I Kongres Nauki Polskiej, Sygn. 13, K. 71–78).

25 Obwohl Kazimierz Ajdukiewicz in einer der Reden erwähnt hat, dass die meisten Autoren der Agenda der Untersektion Marxisten waren: Archiwum PAN, I Kongres Nauki Polskiej, Sygn. 117, K. 91.

26 Siehe, zum Beispiel, den Artikel von Stanisław Lem: (Lem 1951: 2).

27 Kazimierz Ajdukiewicz studierte in Deutschland und besuchte, unter anderen, die Vorlesungen von Edmund Husserl vor dem Ersten Weltkrieg.

28 Archiwum PAN, I Kongres Nauki Polskiej, Sygn. 117, K. 91 a.

29 Archiwum PAN, I Kongres Nauki Polskiej, Sygn. 117, K. 151.

30 Archiwum PAN, I Kongres Nauki Polskiej, Sygn. 117, K. 154; Die Idee der „öffentlichen Debatte“ zwischen „den beiden Weltanschauungen“ spielte, an dieser Stelle, eine wichtige Rolle im Diskurs der Marxisten. Ein ähnliches Argument wurde von Adam Schaff angeführt, der etwas später zu einem der Hauptkritiker der Diskussionsfreiheit wurde, siehe: Archiwum PAN, I Kongres Nauki Polskiej, Sygn. 127, K. 104, 105. In diesem Diskurs wurden alle Widersprüche als Divergenzen zwischen Idealismus und Materialismus bezeichnet.

31 Archiwum PAN, I Kongres Nauki Polskiej, Sygn. 117, K. 154.

32 Archiwum PAN, I Kongres Nauki Polskiej, Sygn. 117, K. 158.

33 Archiwum PAN, I Kongres Nauki Polskiej, Sygn. 117, K. 159.

34 Archiwum PAN, I Kongres Nauki Polskiej, Sygn. 117, K. 134.

35 Archiwum PAN, I Kongres Nauki Polskiej, Sygn. 117, K. 134–135.

36 Archiwum PAN, I Kongres Nauki Polskiej, Sygn. 117, K. 160.

37 Archiwum PAN, I Kongres Nauki Polskiej, Sygn. 117, K. 161.

38 Archiwum PAN, I Kongres Nauki Polskiej, Sygn. 117, K. 162.

39 Rps BUW nr aks 4228, I Kongres Nauki Polskiej, K (2).

40 Rps BUW nr aks 4228, I Kongres Nauki Polskiej, K (2).

41 Rps BUW nr aks 4228, I Kongres Nauki Polskiej, K (3).

42 Rps BUW nr aks 4228, I Kongres Nauki Polskiej, K (3).

43 Rps BUW nr aks 4228, I Kongres Nauki Polskiej, K (3).

44 Rps BUW nr aks 4228, I Kongres Nauki Polskiej, K (3).

45 Rps BUW nr aks 4228, I Kongres Nauki Polskiej, K (3).

46 Die Einleitung für die Übersetzung dieses Buches schrieb Erich Fromm, (Schaff 1965).

47 Rps BUW nr aks 4228, I Kongres Nauki Polskiej, K (3–4).

48 Rps BUW nr aks 4228, I Kongres Nauki Polskiej, K (4).

49 Rps BUW nr aks 4228, I Kongres Nauki Polskiej, K (4).

50 Rps BUW nr aks 4228, I Kongres Nauki Polskiej, K (4).

51 Rps BUW nr aks 4228, I Kongres Nauki Polskiej, K (5).

52 Rps BUW nr aks 4228, I Kongres Nauki Polskiej, K (5).

53 Es ist bemerkenswert, dass Szczurkiewicz auch ein Schüler des „Reaktionärs“ Znaniecki war.

54 Rps BUW nr aks 4228, I Kongres Nauki Polskiej, K (8).

55 Rps BUW nr aks 4228, I Kongres Nauki Polskiej, K (9).

56 Rps BUW nr aks 4228, I Kongres Nauki Polskiej, K (16*8).

57 Rps BUW nr aks 4228, I Kongres Nauki Polskiej, K (25*16); zu Ossowskis Konzeption der „historischen Gesetze“, siehe: Ossowski (1935); Chałubiński (2006).

58 Rps BUW nr aks 4228, I Kongres Nauki Polskiej, K (33*23). Chałasiński publizierte später einen Artikel, in dem er selbst die ganze soziologische Tradition Polens (einschließlich seiner eigenen Werke) als „idealistisch“ und „reaktionär“ bezeichnete (Chałasiński 1951: 75–105).
